# The Role of Emotionality Stigma in Adolescent Mental Health: Measure Development and Call for Systems-Level Change

**DOI:** 10.3390/ijerph21111523

**Published:** 2024-11-16

**Authors:** Hayley D. Seely, Eileen Chen

**Affiliations:** 1 Department of Human Development & Family Studies, Colorado State University, Fort Collins, CO 80523, USA; 2 Denver Health and Hospital Authority, Denver, CO 80204, USA; eileen.chen@dhha.org

**Keywords:** adolescents, measurement, emotion, stigma, mental health, socialization

## Abstract

Youth mental health concerns, including substance abuse, continue to rise. With high co-morbidity rates and a marked lack of representation from diverse groups in study conceptualization, measurement, and implementation, efforts to understand factors impacting youth mental health from a cultural lens are needed. The theory of emotionality stigma posits that many mental health concerns can be understood based on one’s endorsement of emotionality stigma—the experience of stigma around emotions—which manifests within one’s context. Informed by this theory, the current study aimed to adapt and test a measure of emotionality stigma for diverse youth in combined mental health and substance use treatment. Targeted youth focus groups informed the adaptation of the pre-existing Emotionality Stigma Scale for implementation with diverse youth. Using a mixed methods approach, this measure was then tested for relevance, reliability, and validity in an outpatient youth clinic. Patients (*N* = 58, aged 13 to 21) reported their emotionality stigma, values, and attachment as part of routine monitoring. Based on qualitative feedback and quantitative analysis, our results illustrate the reliability and validity of the adapted Emotionality Stigma Scale and the relevance of this new measure for assessing youth mental health concerns and treatment outcomes. Areas for continued research are identified, and recommendations for implementation in conceptualization and treatment are provided.

## 1. Introduction

Despite ongoing efforts to address youth mental health concerns, rates of mood disorders [1] and substance use [2,3] in adolescents continue to rise. These growing mental health concerns are frequently comorbid, resulting in increased risk of additional issues, including impacted academic, social, and emotional functioning [4,5,6,7,8] and life-long symptomatology [9]. These issues are further perpetuated by systemic factors that disproportionately impact diverse youth, including socioeconomic status, experiences of discrimination, and intergenerational trauma [10,11]. However, theory, practice, and research centering culture and youths’ lived experiences are limited [12], and there is a marked lack of support for prevention [13,14] and intervention [15,16] efforts in diverse populations. As such, continued research aimed at informing and improving prevention and intervention efforts that specifically center the lived experiences of diverse youth is needed.

Seely and Mickelson [17] proposed a theory that may be applicable to this work. Specifically, they theorized that a stigma has formed around the experience and expression of emotions. This theory was conceptualized around differences in socialization based on sociocultural factors and centers the role of culture from a theoretical and empirical standpoint [17,18]. Research has found emotionality stigma to be linked with emotion regulation, including suppression, expression, and concealment, as well as mental health, including depression, anxiety, and aggression [19]. 

Central to the conceptualization of emotionality stigma is identifying views of emotionality as an important factor related to mental health concerns that may show up differently based on culture [17,18]. An individual’s views on emotionality, shaped by their cultural values and norms, can influence the ways they process and regulate their emotions [20,21]. Indeed, emotion regulation and processing have both been identified as core factors related to comorbid mental health and substance use concerns [22,23,24]. According to Koole [25], emotion regulation is a process of redirecting and handling one’s emotions. Depending on the person and their experiences, a situation can either result in an increased emotional response (up-regulation) or a decreased emotional response (down-regulation). The benefits of these different regulatory techniques are dependent upon the situation and the purpose of regulation. When used constructively, both up-regulation and down-regulation have been associated with increased well-being [23], an important protective factor for mental health and substance use concerns. In contrast, dysfunction in emotion processing—how an individual takes in their emotional experiences following a disturbing event [26]—is related to mental health and substance use concerns [24].

Difficulty properly regulating emotions along with beliefs about emotionality have been identified as transdiagnostic factors for mental health disorders [19,20,21,22,23,24,25,26,27,28,29,30]. For example, recent research on emotion beliefs has found that the way one views emotions impacts their expression, the use of adaptive regulation techniques, and mental health [27,28,29]. This research aligns with that of emotionality stigma, which has identified a relationship between stigmatized views of emotions, emotion regulation, and mental health concerns [19]. While research on views of emotionality is advancing and expanding into adolescent realms [30], the majority of research in this area has focused on adult populations. Furthermore, emotionality stigma may be uniquely important in understanding mental health as it focuses on generationally and societally transmitted biased views about emotions [17,18], which have the potential to limit help seeking behavior [31] and perpetuate internalized stigma [32]. 

Given the documented role of emotion processing in both mental health and substance use [22,23,24], the application of the theory of emotionality stigma [17,18] could be a meaningful step in improving our understanding of these co-morbid diagnoses in youth. Furthermore, as emotionality stigma accounts for context-dependent judgements and prejudices that may underly emotion expression [18], application in clinical spheres may improve treatment processes and outcome monitoring from a culturally informed, theoretical lens. However, although the underpinnings of emotionality stigma are widely applicable, the theory has only been empirically tested in adult populations [19]. The research supported the three theorized dimensions of emotionality stigma—stigma endorsement, stigma resistance, and differential treatment—and illustrated the reliability and validity of the scale in a large, diverse adult sample. Adapting this measure to be used with adolescent populations impacted by co-morbid mental health and substance use concerns has research implications (e.g., improving our understanding of the role of emotionality stigma as a risk factor related to youth mental health and substance use), as well as clinical utility (e.g., informing treatment and progress monitoring).

Given the vast need for effective mental health treatment, as well as culturally responsive efforts and measurement, the current study sought to (a) adapt a measure of emotionality stigma for youth and (b) apply and test this measure within an adolescent treatment setting specialized in providing care for youth presenting with co-morbid mental health and substance use concerns. Based on these two aims, we hypothesized that the construct of emotionality stigma would hold within the adolescent population and that an adapted measure informed by adolescent focus groups would be positively correlated with the original measure and negatively correlated with measures of well-being (e.g., an adolescent’s values and level of attachment). We also proposed that we would find the same three underlying domains of emotionality stigma (e.g., stigma endorsement, stigma resistance, and differential treatment) and that the adapted scale would meet reliability standards at the subscale and total scale levels. 

## 2. Materials and Methods

Outcomes data are regularly collected as part of the program’s ongoing quality improvement procedures. These data are also used to inform programmatic and clinical changes to enhance the delivery of services. This evaluation is part of a larger non-randomized, retrospective chart review designed to determine whether interventions aimed at reducing barriers to treatment are associated with an increase in engagement in, retention, and completion of therapeutic programming amongst historically underserved adolescents. This evaluation was reviewed and approved by the Colorado Multiple Institutional Review Board (COMIRB # 23-2401). 

Patients are referred to the Substance Treatment, Education, and Prevention (STEP) program through community partners, including hospitals, care networks, physicians, and self-referrals. Upon completing an intake to the STEP program, intake therapists complete an assessment to determine the patient’s ASAM Criteria Level. The ASAM Criteria are a unified set of standards for determining patient admission, continued service, and transfer criteria by assessing patient needs in relation to six dimensions (e.g., Acute Intoxication and/or Withdrawal Potential; Biomedical Conditions and Complications; Emotional, Behavioral, or Cognitive Conditions and Complications; Readiness to Change; Relapse, Continued Use, or Continued Problem Potential; and Recovering/Living Environment [33]). 

Only patients that qualified for ASAM Level 2.1 or higher (*N* = 58, aged 13 to 21) were included in this study, as these patients receive a higher level of care and thus receive more extensive routine monitoring than patients at a lower ASAM level. Standard care for ASAM Level 2.1 patients includes individual therapy, case management, an optional group curriculum, medication management, and parent support/consultation. Primary substance use clinical presentations include, but are not limited to, alcohol use disorder, cannabis use disorder, cocaine use disorder, methamphetamine use disorder, and opioid use disorder. Co-occurring psychiatric disorders include, but are not limited to, adjustment disorder, generalized anxiety disorder, major depressive disorder, posttraumatic stress disorder, and social anxiety disorder. Patients consented to treatment at intake and continued to assent to treatment by continuing to present to and engage in therapeutic interventions. No patients who qualified for ASAM Level 2.1 were excluded. 

As part of routine monitoring for patients qualified for ASAM Level 2.1, clinicians asked patients to complete a questionnaire at the beginning of treatment, as well as 4 and 8 weeks into treatment to gage their progress. Included as part of this questionnaire are the Emotionality Stigma Scale (ESS) [19] and two measures of well-being—the Valuing Questionnaire (VQ) [34] and the Adolescent Attachment Questionnaire (AAQ) [35]. The ESS is a 30-item measure created to assess an individual’s emotionality stigma across three dimensions: stigma endorsement, stigma resistance, and differential treatment. Patients reported the extent to which they agreed with each statement on a 4-point Likert Scale (1 = strongly disagree; 4 = strongly agree). This scale has met reliability and validity standards in an adult population [19]. The VQ consists of 10 self-report items that assess the consistency with which an individual has been living in line with their values. Participants responded using a 7-point Likert Scale (0 = not true at all; 6 = completely true), and items were summed, with higher scores indicating more alignment with values. The VQ met reliability standards in our sample with an internal consistency of α = 0.879. The AAQ is designed to measure an adolescent’s level of attachment to their primary caregiver. Using a 5-point Likert Scale (1 = strongly disagree; 5 = strongly agree), participants rated their level of agreement on 9 items. These items were summed, resulting in adequate internal consistency (α = 0.855).

Given that the Emotionality Stigma Scale (ESS) had not been tested within an adolescent population, adolescent focus groups were utilized to adapt the measure. These focus groups were voluntary and occurred in addition to standardized treatment. Participants assented to participate in these focus groups and were permitted to leave the group at any time. We held focus groups multiple times throughout a 3-month period. These groups ranged in size from 5–8 youth. Youth who participated in the voluntary focus groups were representative of the study population in terms of level of care, race, ethnicity, age, and socioeconomic status. During the focus groups, participating youth collaboratively identified scale items that were relevant and irrelevant to their lived experience, made edits to the language used in the items, and provided feedback regarding their understanding of the meaning of each item. Based on focus group feedback, adjustments were made to the scale, and the adapted scale was re-reviewed. This was an iterative process that resulted in the 17-item Emotionality Stigma Scale for Adolescents (ESS-A) tested herein. The items adapted and retained in the ESS-A were theoretically mapped onto the three dimensions of emotionality stigma [19], with items reflecting stigma endorsement (e.g., “Some emotions should not be expressed”), stigma resistance (e.g., “Emotions make life more meaningful”), and differential treatment (e.g., “People who express their emotions are judged”). 

Prior to hypothesis testing, we investigated the diversity of our sample using descriptive statistics of age, gender, race, ethnicity, need for transportation assistance, and insurance status. The factor structure was then assessed using exploratory factor analysis (EFA). According to EFA sample recommendations [36,37], a minimum sample size of 50–55 would be needed to conduct the intended analyses based on estimated high factor loadings and communities between items and the ratio of variables to factors. EFAs were conducted for both the ESS and the ESS-A. For the ESS, we conducted EFA only to check the factor structure in an adolescent population. Therefore, we utilized an EFA with Promax rotation with three fixed factors defined according to emotionality stigma theory [17]. We also considered the qualitative data collected during youth focus groups by identifying trends in the feedback for the ESS and the adapted ESS-A. For the quantitative development of the ESS-A, we conducted two separate EFAs with Promax rotation by comparing the statistically driven factor solutions with one based on emotionality stigma theory [17]. The first provided an eigenvalue to define the number of factors, whereas in the second EFA, the three theorized factors were specified. With these different analyses as evidence, we used the Kaiser criterion, a scree plot, theory, and parallel analysis to define item exclusion and ultimately identify the best-fitting factor solution. Reliability was assessed using internal consistency analyses of the items and correlations of subscale and total scale scores across two timepoints. To test validity, correlations of the ESS-A with the original ESS and adolescent well-being measures (VQ and AAQ) were also conducted. 

## 3. Results

### 3.1. Sample Descriptions

The sample consisted of 58 adolescents aged 13–21 (M = 15.79, SD = 1.65) in combined mental health and substance use treatment. The majority of participants identified as men (62.1%), with women (32.8%) and transgender and non-binary adolescents (5.2%) also represented. Our sample was diverse, with participants identifying as Hispanic (34.5%), Black (6.9%), American Indian or Alaskan Native (3.4%), Multiracial (15.5%), and White (39.7%). Most participants required transportation assistance to attend therapy sessions (55.2%) and were either insured through Medicaid or uninsured (63.8%). 

### 3.2. Exploratory Factor Analyses and Internal Consistency for ESS

In support of the hypotheses, the construct represented in the original ESS remained consistent overall in the adolescent sample, but it illustrated notable differences, further supporting the need for an adapted measure. Specifically, although the 3-factor solution produced results that mapped onto the three dimensions of emotionality stigma [17] and followed a similar structure to that supported in an adult sample [19], multiple items loaded onto different dimensions (Table 1), and the overall fit of the factor solution was inadequate (KMO = 0.459). Furthermore, reliability statistics illustrated less internal consistency of the dimension items than supported in past research [19], with internal consistencies of 0.732, 0.693, and 0.866 for stigma endorsement, stigma resistance, and differential treatment, respectively. 

### 3.3. Qualitative Feedback on the ESS and the ESS-A

Through the course of the utilized focus groups, participating youth provided qualitative feedback on the theory of emotionality stigma as well as the original measure and the collaboratively adapted measure. Overall, youth found the concept of emotionality stigma to be relevant, sharing experiences in their lives and families in which emotionality stigma has impacted them. Participating youth also shared their own endorsement of stigma around specific emotions, identifying that some emotions feel “safer” to express. 

When asked to provide feedback on the original ESS, youth identified items that were relevant to them (e.g., “Emotions have made my life more meaningful”, “People ignore people who are emotional or take them less seriously just because they are emotional”) as well as items that felt less relevant to their lived experience (e.g., “Emotional people cannot live a good, rewarding life”, “Emotions make me unique”). In addition, youth identified specific words as confusing or unhelpful (e.g., “patronize”, “inadequate”, “inferior”) and shared that the overall length of the ESS made it difficult to maintain attention and effort. 

Following the iterative process of creating the ESS-A, youth provided positive feedback, appreciating the decreased length, the simplified language and sentence structure, and the increased application to their experiences. While some groups provided feedback that the measure was still too long, in a collaborative discussion about what items to remove, it was determined that the remaining items were theoretically important to the purpose of the measure. Therefore, the 17 items were retained for the purpose of this evaluation, with empirically supported item reduction as a goal.

### 3.4. Exploratory Factor Analyses and Internal Consistency for ESS-A

Where the original ESS illustrated some discrepancies from the original measurement findings, the adapted ESS-A supported a 3-factor solution aligned with the three theoretical dimensions—stigma endorsement, stigma resistance, and differential treatment. Although the Kaiser criterion suggested a 4-factor solution, the scree plot (Appendix B) and the theory supported 3 factors. Furthermore, in the 4-factor solution, the 4th factor had an eigenvalue of 1.128, only accounting for 6.63% of the variance in the model (Appendix B). 

Given the conflicting support for a 4-factor solution and a 3-factor solution, the factor loadings of these solutions were compared (Table 2). Overall, the 4-factor solution and the 3-factor solution were highly consistent, with all but one item (item 6: “Some emotions should not be expressed”) loading onto the same 3 factors. In the 4-factor solution, this item loaded onto the 4th factor, suggesting that this item may not capture the intended meaning and may not meaningfully contribute to the overall scale. This was further supported in the 3-factor solution, where item 6 displayed low loadings and high cross-loadings. Based on these statistical findings and considerations of the theoretical assumptions of emotionality stigma, this item was excluded. Although in both factor solutions another item (item 17: “I wish I could get rid of my emotions”) was flagged due to having high cross-loadings, as the dimensions of emotionality stigma are theorized to correlate and this item is theoretically relevant, it was retained in the final model. After item reduction procedures in which item 6 was removed, the EFA supported a 3-factor solution based on the Kaiser criterion, the scree plot, and the theory, with the factors accounting for 69.73% of the variance in the model. 

The final Emotionality Stigma Scale—Adolescents (see Appendix A.2) consisted of 16 items with subscales representing the three dimensions of emotionality stigma: the stigma endorsement subscale (3 items, α = 0.688), the stigma resistance subscale (5 items, α = 0.866), and the differential treatment subscale (8 items, α = 0.880). The total scale also met reliability standards, with an internal consistency of α = 0.838.

### 3.5. Correlations Between ESS-A and Related Constructs

As hypothesized, the ESS and the adapted ESS-A were correlated in our sample at both the subscale and the total scale level (Table 3). Furthermore, the ESS-A showed consistency over time, with highly correlated scores across two timepoints (Table 4). Illustrating convergent validity, as hypothesized, the ESS-A was negatively correlated with adolescents’ reports on the Valuing Questionnaire (r = −0.66, *p* < 0.001) and the Adolescent Attachment Questionnaire (r = −0.39, *p* = 0.035). 

## 4. Discussion

The role of emotion processing in both mental health and substance use [24] and the vast need for culturally informed prevention and intervention efforts [13,14,15,16] are well-documented. Thus, we aimed to apply the theory of emotionality stigma [17,18] to a diverse adolescent population to better understand the function of emotionality stigma in mental health and substance use comorbidity for youth. Informed by adolescent focus groups, we adapted the ESS [19] for adolescents in combined mental health and substance use treatment. As hypothesized, the construct of emotionality stigma remained relevant in the diverse adolescent population based on both youth reports and quantitative evidence. Although the EFA of the ESS illustrated some inconsistency in the factor structure from the structure found with adult populations [19], the adapted ESS-A followed the theorized 3-factor structure, with factors representing stigma endorsement, stigma resistance, and differential treatment. 

In testing the ESS-A for reliability and validity, the results suggest that the measure meets the standards. Internal consistencies ranged from 0.688 to 0.880, suggesting that the items within each subscale were reliably assessing the same dimension. Across time, adolescent reports on the measure remained consistent, providing support for test–retest reliability. Regarding validity, the adapted measure was correlated with the original measure (ESS), illustrating construct validity. We also found the ESS-A to be negatively correlated with measures of well-being as hypothesized, suggesting convergent validity. 

Overall, the results provide preliminary support for the applicability of emotionality stigma in diverse adolescent populations. The adapted measure of ESS-A functioned well within the sample population and met reliability and validity standards at the subscale and the total scale levels. Given these findings, integrating emotionality stigma into future research through the ESS-A is warranted. Theoretically, emotionality stigma may have vast, transdiagnostic applications to mental health and substance use treatment [17,18], which have gained preliminary empirical support in adult populations [19] and, through this work, adolescent populations. Future research should continue to explore the construct of emotionality stigma in both populations, including investigating it as a mechanism of change in treatment settings. 

Furthermore, given the diversity of our sample, emotionality stigma appears to be a prevalent construct across various cultures. This suggests that the impact of emotionality stigma on mental health and substance use disorders may be universal, while the ways in which it manifests and interacts with cultural norms can differ significantly. Thus, understanding these variations is crucial for developing culturally responsive interventions that address this cross-culturally relevant construct. Given the relevance of emotionality stigma across diverse populations, future research should continue investigating how emotionality stigma functions within specific cultural contexts. Another important research aim will be to explore ways in which emotionality stigma contributes to the development of mental health and substance use disorders, as well as its impact on treatment intervention. Such research could offer valuable insights into tailoring prevention and treatment efforts in ways that are both culturally informed and effective. 

In addition to the application of this work to future research and practice, the relevance of emotionality stigma in both adolescent and adult populations [19] has important ramifications for policy and systems-level change. Emotionality stigma is theorized to arise from the emotion socialization process, shaped by the unique expectations of emotionality in different groups [38,39,40,41]. Thus, movements to dismantle current emotionality norms and to create systems in which emotion expression is safe for diverse groups have the potential to actively diminish emotionality stigma. While this preliminary research calls for systems-level change, community-level policies and resources to increase access to care, as well as treatment strategies that integrate the role of emotionality stigma and relevant contextual factors, are tangible first steps. 

Our study has many strengths, including the diversity of our sample, the co-creation of the adapted ESS-A measure through collaborative adolescent focus groups, and the transdiagnostic orientation. This study also has limitations that are important to consider in interpreting our findings. Most notably, given the context of our sample, we have a relatively small sample size, which could increase the likelihood of both type 1 and type 2 errors. Relatedly, although we correlated the ESS and ESS-A, which is an important aspect of assessing construct validity, these surveys were not completed at the same timepoint due to patient time limitations. Therefore, it may be that the correlations between these two measures would shift if they were administered in the same survey. Given that our sample was in combined mental health and substance use treatment, the effect of treatment effects on reports of emotionality stigma over time must also be considered, as well as the generalization to non-clinical adolescent populations. 

Considering our findings along with the limitations of this study, it is recommended that future research investigate the applicability of emotionality stigma and the ESS-A in larger clinical and non-clinical adolescent populations. Following additional empirical support for this measure and emotionality stigma as a transdiagnostic construct, efforts to embed emotionality stigma into intervention and prevention efforts may increase the effectiveness of such efforts for diverse youth and may provide an additional mechanism of change that can be monitored throughout therapy via the ESS-A. 

## 5. Conclusions

Emotion processing is a critical mechanism associated with mental health and substance use [24], two of the most prevalent issues impacting youth today [1,2,3]. Co-morbid mental health and substance use concerns are highly impactful on an individual’s lived experience [4,5,6,7,8,9] and disproportionately effect diverse youth due to systemic factors, including socioeconomic status, experiences of discrimination, and intergenerational trauma [10,11]. Applying culturally informed, transdiagnostic theories, such as the theory of emotionality stigma [17,18], may be a meaningful pathway for improving current prevention and intervention efforts. The current study provides preliminary support for the theory of emotionality stigma as a meaningful construct and the ESS-A as a relevant measure for diverse adolescent samples, including, specifically, adolescents in combined mental health and substance use treatment. This study provides a pathway for additional research to better understand the function of emotionality stigma in mental health and substance use treatment with the goal of advancing our understanding of emotionality stigma as a treatment mechanism, developing culturally informed treatment modalities, and inspiring movements to increase the safety of emotionality at the community and societal levels. 

## Figures and Tables

**Table 1:** EFA factor loadings for ESS based on 3-factor solution.

Item	1	2	3
22. Emotional people don’t socialize as much as they used to because being emotional might make them look or behave “weird.”	**0.827**	0.129	−0.107
23. Negative stereotypes about emotionality keep emotional people isolated from the “normal” world.	**0.694**	0.221	−0.100
25. Being around people who aren’t emotional makes emotional people feel out of place or inadequate.	**0.638**	−0.183	−0.455
21. Emotional people don’t talk about themselves much because they don’t want to burden others with their emotionality.	**0.609**	0.144	−0.155
9. People feel embarrassed or ashamed when they are emotional.	**0.601**	0.139	−0.243
24. Emotional people stay away from social situations in order to protect their family or friends from embarrassment.	**0.593**	−0.220	0.116
19. People often patronize those who are emotional, just because they are emotional.	**0.578**		0.347
26. Emotional people avoid getting close to people who aren’t emotional to avoid rejection.	**0.562**		
18. People ignore people who are emotional or take them less seriously just because they are emotional.	**0.524**		0.380
13. When people are emotional, they need others to make most decisions for them.	**0.438**	0.197	−0.437
17. Others think that people who are emotional can’t achieve much in life.	**0.420**	−0.211	0.211
11. People who are emotional feel inferior to others who are not emotional.	**0.276**	−0.227	0.228
15. Emotional people can’t contribute anything to society.		**0.758**	0.146
28. Emotional people can have a good, fulfilling life, despite their emotionality. *	0.269	**0.746**	0.195
29. Emotional people make important contributions to society. *		**0.612**	0.158
3. Emotions allow people to be imaginative and/or creative. *	−0.148	**0.568**	0.224
6. Emotions makes me unique. *		**0.539**	0.160
14. Emotional people cannot live a good, rewarding life.	0.338	**0.534**	
1. Emotions let people think in interesting and insightful ways. *	−0.193	**0.440**	−0.163
12. Stereotypes about emotionality are valid.		**0.413**	
27. In general, emotional people are able to live life the way they want to. *	0.272	**0.409**	
30. Living with emotions makes people tough. *	−0.294	**0.317**	
10. People who are emotional should be disappointed in themselves for being emotional.		**0.291**	0.152
7. People should feel out of place in the world if they are emotional.	0.114	**0.236**	−0.110
5. Emotions are a source of weakness.	−0.237		**0.585**
8. Emotions spoil peoples’ lives.		0.183	**0.537**
4. Emotions have made my life more meaningful. *		0.222	**0.513**
2. Emotions make people less productive.		0.184	**0.500**
16. People discriminate against individuals who are emotional.	0.375	−0.328	**0.470**
20. Nobody would be interested in getting close to someone who is emotional.		0.185	**0.400**

Note: Items are listed in the order indicated based on the 3-factor solution. Bolded values represent those loading onto each factor. Underlined items indicate items that are loading differently than the factor solution in adult populations [19]. * = reverse-coded item.

**Table 2:** EFA factor loadings and fit criteria for ESS-A, with the 3-factor solution compared to the 4-factor solution.

Item	3-Factor Solution			4-Factor Solution			
	1	2	3	1	2	3	4
14. People who express their emotions are judged.	**0.883**			**0.883**			
12. Society takes emotional people less seriously.	**0.817**	−0.219		**0.817**	−0.204		
8. Emotions make life harder.	**0.799**		0.245	**0.782**		0.283	0.292
16. I feel the need to hide my emotions.	**0.768**			**0.803**		0.202	−0.102
13. There is a societal message that emotions are bad.	**0.760**	−0.391		**0.738**	−0.390	−0.121	0.198
17. I wish I could get rid of my emotions.	**0.715**	0.673	−0.218	**0.669**	0.582		0.133
15. Emotions make one feel less than others.	**0.598**	0.189		**0.568**	0.175		0.210
6. Some emotions should not be expressed.	0.311		0.207	0.250		0.127	**0.756**
2. Emotions make you creative. *		**0.856**	0.106		**0.835**	0.345	0.159
1. Emotions make you insightful. *	−0.143	**0.757**	0.132	−0.174	**0.754**	0.280	0.245
3. Emotions make life more meaningful. *		**0.712**			**0.723**	0.337	
5. Emotions make you unique. *		**0.707**	−0.357		**0.636**	0.391	0.258
4. Emotions are important. *		**0.599**	0.266		**0.592**	−0.106	−0.131
11. There are stereotypes about being emotional.	**0.454**	−0.516		**0.476**	−0.504	−0.161	−0.150
7. Some people should not be emotional.		−0.188	**0.859**			**0.728**	0.133
9. It is weird when people express emotions in public.	0.227		**0.642**	0.258	0.265	**0.651**	0.174
10. I withdraw from people when they are too emotional.		0.179	**0.519**	0.108	0.314	**0.648**	−0.105

Note: Items are listed in the order indicated based on the 3-factor solution. Coefficients below 1 were suppressed and are not reported here. Bolded values represent those loading onto each factor. Underlined values indicate cross-loadings with a difference between loadings of < 0.1. Underlined items indicate items considered for exclusion based on low loadings, cross-loadings, or theoretical irrelevance; these items were excluded in the final model. * = reverse-coded item.

**Table 3:** Bivariate correlations of ESS and ESS-A.

Measure	1	2	3	4	5	6	7	8
1. ESS: Stigma Endorsement	__							
2. ESS: Stigma Resistance	0.27 *	__						
3. ESS: Differential Treatment	0.13	−0.23	__					
4. ESS: Total	0.67 ***	0.35 **	0.72 ***	__				
5. ESS-A: Stigma Endorsement	0.45 *	0.19	0.20	0.45 *	__			
6. ESS-A: Stigma Resistance	0.13	0.19	0.09	0.20	0.38 *	__		
7. ESS-A: Differential Treatment	0.34	−0.11	0.62 ***	0.57 ***	0.27	−0.04	__	
8. ESS-A: Total	0.44 *	0.04	0.58 ***	0.65 ***	0.63 ***	0.44 *	0.85 ***	__
Mean	18.37	15.28	29.72	63.37	5.52	10.10	21.06	36.68
Standard Deviation	4.68	4.03	7.62	10.04	2.08	2.68	5.92	7.50

Note: * *p* < 0.05, ** *p* < 0.01, *** *p* < 0.001.

**Table 4:** Bivariate correlations of ESS-A across two timepoints.

Time	Measure	1	2	3	4	5	6	7	8
1	1. ESS-A: Stigma Endorsement	__							
	2. ESS-A: Stigma Resistance	0.28 *	__						
	3. ESS-A: Differential Treatment	0.27	−0.04	__					
	4. ESS-A: Total	0.63 ***	0.44 *	0.85 ***	__				
2	5. ESS-A: Stigma Endorsement	0.62 **	−0.21	0.33	0.39	__			
	6. ESS-A: Stigma Resistance	0.19	0.71 ***	−0.45	−0.02	−0.22	__		
	7. ESS-A: Differential Treatment	0.28	0.23	0.65 **	0.71 ***	0.29	−0.26	__	
	8. ESS-A: Total	0.50*	0.42	0.53 *	0.76 ***	0.45	0.07	0.92 ***	__
	Mean	5.52	10.10	21.06	36.68	5.11	10.72	22.22	38.06
	Standard Deviation	2.08	2.68	5.92	7.50	1.75	2.40	6.10	6.53

Note: * *p* < 0.05, ** *p* < 0.01, *** *p* < 0.001.

**Figure 1:**
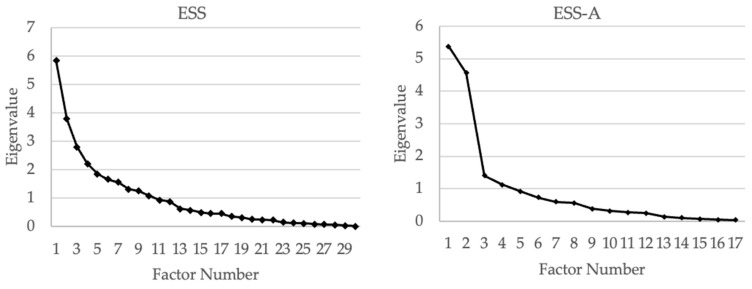
EFA scree plots.

## Data Availability

The data and the codes can be accessed by emailing the corresponding author.

## Appendix A.

### Appendix A.1. Emotionality Stigma Scale [19]

Please indicate to what extent you agree or disagree with the following statements (1 = strongly disagree; 4 = strongly agree).

1. Emotions let people think in interesting and insightful ways. *
2. Emotions make people less productive.
3. Emotions allow people to be imaginative and/or creative. *
4. Emotions have made my life more meaningful. *
5. Emotions are a source of weakness.
6. Emotions makes me unique. *
7. People should feel out of place in the world if they are emotional.
8. Emotions spoil peoples’ lives.
9. People feel embarrassed or ashamed when they are emotional.
10. People who are emotional should be disappointed in themselves for being emotional.
11. People who are emotional feel inferior to others who are not emotional.
12. Stereotypes about emotionality are valid.
13. When people are emotional, they need others to make most decisions for them.
14. Emotional people cannot live a good, rewarding life.
15. Emotional people can’t contribute anything to society.
16. People discriminate against individuals who are emotional.
17. Others think that people who are emotional can’t achieve much in life.
18. People ignore people who are emotional or take them less seriously just because they are emotional.
19. People often patronize those who are emotional, just because they are emotional.
20. Nobody would be interested in getting close to someone who is emotional.
21. Emotional people don’t talk about themselves much because they don’t want to burden others with their emotionality.
22. Emotional people don’t socialize as much as they used to because being emotional might make them look or behave “weird.”
23. Negative stereotypes about emotionality keep emotional people isolated from the “normal” world.
24. Emotional people stay away from social situations in order to protect their family or friends from embarrassment.
25. Being around people who aren’t emotional makes emotional people feel out of place or inadequate.
26. Emotional people avoid getting close to people who aren’t emotional to avoid rejection.
27. In general, emotional people are able to live life the way they want to. *
28. Emotional people can have a good, fulfilling life, despite their emotionality. *
29. Emotional people make important contributions to society. *
30. Living with emotions makes people tough. *

* Reverse-coded items. Sum scores are used to calculate the subscales and the total scales. Items 2, 5, 7, 8, 10, 12, 13, 14, 15, and 20 make up *Stigma Endorsement*. Items 1, 3, 4, 6, 27, 28, 29, and 30 make up *Stigma Resistance.* Items 9, 11, 16, 17, 18, 19, 21, 22, 23, 24, 25, and 26 make up *Differential Treatment.*

### Appendix A.2. Emotionality Stigma Scale—Adolescents

Please indicate to what extent you agree or disagree with the following statements (1 = strongly disagree; 4 = strongly agree).

1. Emotions make you insightful *
2. Some people should not be emotional
3. Emotions make you creative *
4. Emotions make life more meaningful *
5. I withdraw from people when they are too emotional
6. Emotions are important *
7. It is weird when people express emotions in public
8. Emotions make you unique *
9. Emotions make life harder
10. There are stereotypes about being emotional
11. Society takes emotional people less seriously
12. There is a societal message that emotions are bad
13. Emotions make one feel less than
14. People who express their emotions are judged
15. I feel the need to hide my emotions
16. I wish I could get rid of my emotions

* Reverse-coded items. Sum scores are used to calculate the subscales and the total scales. Items 2, 5, and 7 make up Stigma Endorsement. Items 1, 3, 4, 6, and 8 make up Stigma Resistance. Items 9–16 make up Differential Treatment.

## Appendix B.

**Table A1:** EFA Kaiser criterion.

**ESS**					
	**Initial Eigenvalue**		**Extraction SS Loadings**		**Rotation SS** **Loadings**
Factor	Total	Variance	Total	Variance	Total
1	5.857	19.522	5.321	17.736	4.930
2	3.785	12.617	3.163	10.544	3.998
3	2.802	9.339	2.153	7.177	2.847
**ESS-A**					
	**Initial Eigenvalue**		**Extraction SS Loadings**		**Rotation SS** **Loadings**
Factor	Total	Variance	Total	Variance	Total
1	5.382	31.661	5.070	29.824	4.597
2	4.565	26.854	4.252	25.013	4.391
3	1.413	8.314	0.967	5.689	3.299
4	1.128	6.634	0.759	4.462	2.072

Note: SS = sums of squared.
